# Supplementary material for: Kemeny-based testing for COVID-19
Source: PLoS One. 2020 Nov 19;15(11):e0242401. doi: 10.1371/journal.pone.0242401 (PMC7676669; doi:10.1371/journal.pone.0242401)
Supplement: Attachment — Submitted filename: Response to Reviewers.pdf [file pone.0242401.s001.pdf]

Revision of manuscript PONE-D-20-23601

Dear Editor-in-Chief,

Please find enclosed the revision of manuscript PONE-D-20-23601 “Kemeny-based testing for COVID-19”.

Our responses to the comments of the Reviewers have been incorporated into the revised manuscript. Also, point by point responses to the comments are reported below (Reviewers’ comments are reported in black, while our responses are in blue).

We hope that the paper is a significant improvement on the previous one and can now be accepted for publication.

Yours sincerely,

Emanuele Crisostomi

(on behalf of my co-authors)

Reviewer #1: This is a very good work.

It points out several important points in the analysis of the infection spread prevention in terms of testing. The paper is very well written solid and interesting. The first idea one can have of targeting the highly connected nodes can indeed be very misleading.
I definitely recommend publication. I do not have essential change to suggest. Since I was very interested I add some minor comments/considerations. The Authors may decide to consider in the final version or in future work (or disregard them).

We thank the Reviewer for the general very positive feedback. Below our responses to the specific comments.

As a pure academic considerations, the proposed index can be referred to a group of nodes.
I mean there can be that two nodes (this is not quite realistic) that have very high Kemeny indicators, but if one of them is removed then the index of the other immediately drops. This is the case in which two communities are connected by two consecutive nodes, so once one of these is removed the other becomes absolutely 'insignificant'. So if k nodes can be removed, then only one of these two should be removed. Accordingly one can think to the k-index which is computed by considering the effect of removing k nodes. This leads clearly to a large complexity (combinatorial) in the evaluation.

The Reviewer is raising an interesting point. It is possible to devise "ad hoc" rules to improve the basic functioning of the Kemeny-based indicator to address some possible limit cases (one being the example provided by the Reviewer). For instance, one possible improvement (which, if we understand correctly, is different from the one suggested by the Reviewer) is to choose the single node with highest ranking; remove it from the network; and then rank all nodes again according to the new network (and repeat this procedure until the list of individuals who should be tested is complete).

Such a procedure would be more computationally demanding, but would actually improve not only the ranking of the Kemeny-based indicator, but of all the other indicators as well.

In our work, we decided to simultaneously compute the most critical nodes according to all indicators, to maintain the presentation and the computations simple, while preserving the fairness of the comparison.

In the revised manuscript we have added a remark to clarify that an alternative way of choosing the most critical nodes could be adopted.

Another important issue for the future is how one can compute, possibly in an approximate way, the suggested indicator for large network.

This is also an interesting point. According to the final application of our proposed methodology, one may be interested in obtaining the solution in a very short time. Since (one way of) computing

the Kemeny constant requires the computation of all the eigenvalues of the transition matrix (see Equation (4)), a straightforward approximation of the indicator may only involve the second largest eigenvalue modulus (SLEM) (or the first few eigenvalues for a more accurate approximation).

We however prefer to leave this problem as a future work, together with the somewhat related problem of obtaining a distributed estimate of the indicator.

I would spend more words to the case of edge Kemeny index (achieved by removing an edge).

We assume that here the Reviewer was actually referring to the Kemeny index achieved by removing a node (and not an edge). In this case, in the revised manuscript we now anticipate the more formal definition to the previous section, as suggested by the same Reviewer in a later question.

If the Reviewer was truly referring to the case of the Kemeny constant *after removing an edge*, we refer to an earlier published paper of some of the authors where the problem of estimating the change of the Kemeny constant by varying some non-diagonal entries of the transition matrix is address [1, Equation (14)]. However, while node removal makes perfect sense in the application of interest here (as it corresponds to quarantine), edge removal has a less straightforward interpretation here (prevent a single specific contact between two individuals from occurring?), and the theory developed in [1] has not been used in the current work.

[1] E. Crisostomi, S. Kirkland and R. Shorten, “A Google-like model of road network dynamics and its application to regulation and control”, International Journal of Control, vol. 84, no. 3, pp. 633-631.

Finally, as an opinion, I think that these type of considerations will be more important in view of future vaccination strategies (when a vaccine will be available) rather than in testing.

We agree with the Reviewer that the methodology can be perfectly used for vaccination strategies in general, independently also from the specific COVID-19 case.

MINOR

- Kemeny constant tends to infinity I would just say Kemeny constant becomes infinite

  The Kemeny constant had been originally defined for fully connected graphs, so, in principle, some mathematicians may argue that it should never occur that it may become infinite. That is why we tried to mitigate the sentence that “tends” to infinity.

- The index Kemeny indicator is defined in a very colloquial way in the simulation section as follows: “For this purpose, we quantify the important of each single node as the corresponding value of the Kemeny constant of the graph obtained from the original graph by removing such a node, and for simplicity we shall denote it as the Kemeny indicator”. I would anticipate this as a more formal definition in the previous section.

As from an earlier comment of the same Reviewer, we have now revised this aspect as suggested.

Reviewer #2: This paper proposes an interesting method, based upon the so-called Kemeny constant, to determine which individuals to test, in case of limited resources for testing. The results are promising although the results are based on a limit number of simulations and relatively small networks.

We thank the Reviewer for the comment, and we understand the concern regarding the limited size of the used networks.

In principle, we have used small networks to better explain the main idea of the proposed approach (i.e., testing bridges between communities instead of testing nodes with high degrees, which may very give rise to new outbreaks in previously healthy communities).

In our experience, if networks are created according to the same procedure outlined in our manuscript (i.e., a community-based structure with modularity 0.8), then similar results are obtained in larger networks as well. For instance, the example below regards a network with 25 communities of 40 individuals (i.e., 10^3 individuals overall), created with the procedure taken from the literature (the "Benchmark" graph from paper [25] of our list of references), starting from an initial average degree of 4, with 4 initially infected individuals. We assume that 100 individuals (10%) are tested every day.

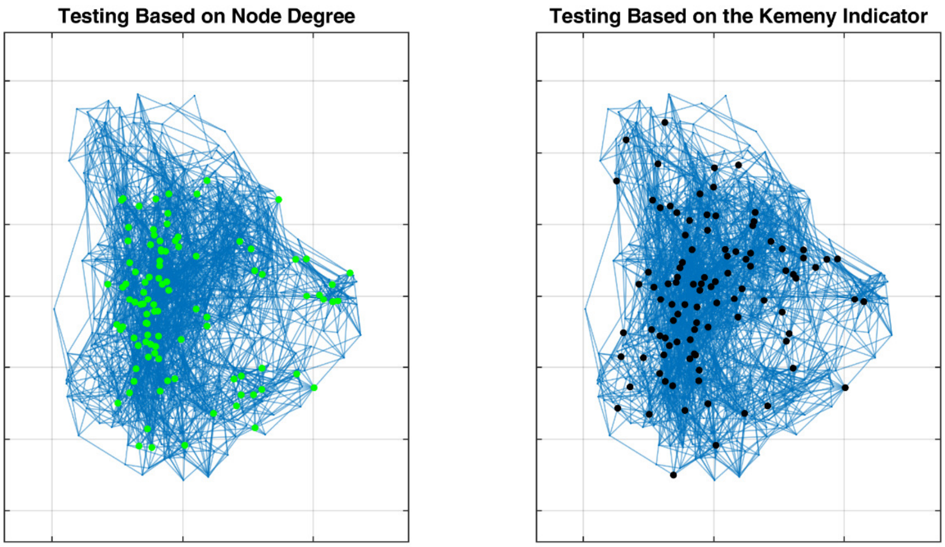

Fig. 1: *Tested individuals in a population of 1000 individuals (split in 25 communities of 40 individuals each).*

Then, the figure on the left shows the individuals tested according to the "high degree" indicator, and the figure on the right shows the individuals tested according to the "Kemeny indicator". Given the large number of individuals, and the large number of connections, it is hard to appreciate the

criterion underlying the choice of individuals that are chosen to be tested (which is the main reason why we have shown examples with fewer nodes and edges). In this network of larger size, similar results are obtained again, as shown in Fig. 2 where the peak number of infected individuals is reduced from 180 to about 110 individuals if testing is based on the proposed Kemeny indicator.

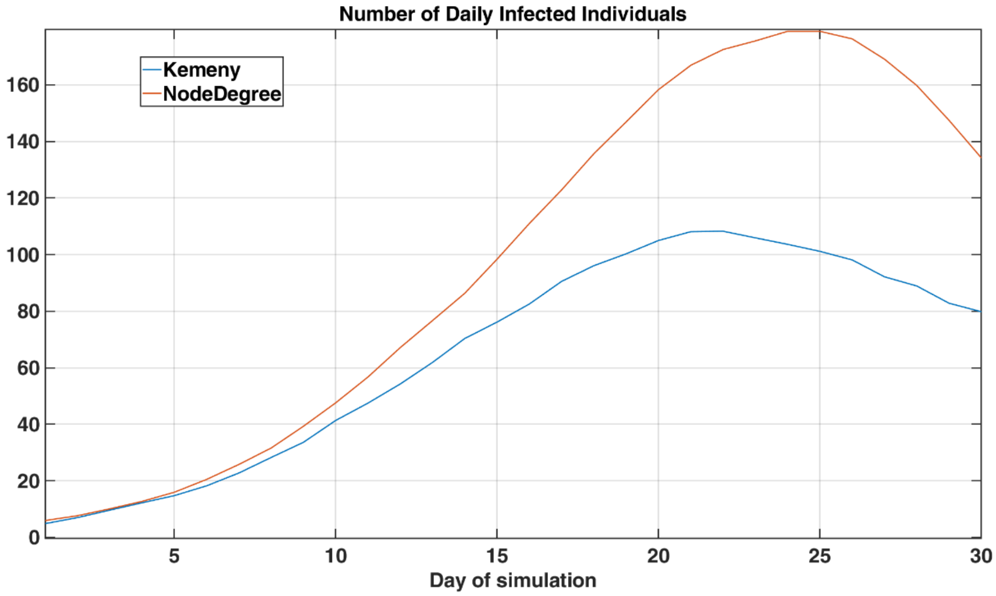

Fig. 2: *Number of daily infected individuals in the network of 1000 individuals, depending on the criterion chosen for testing.*

While graph theory and Markov chains have been widely employed for very large-scale problems (most notably, think of PageRank for all web pages), still when the number of individuals grows of further orders of magnitude (say, millions of individuals), then the main problem becomes that of estimating the Kemeny indicator in a quick, and yet accurate, way. While simple off-the-shelf solutions already exist - e.g., think of the problem of just estimating the second largest eigenvalue modulus SLEM) – we leave this for a future work (e.g., to estimate the accuracy of approximated approaches).

Some detailed comments:

- It seems the order of the Figures doesn't correspond with the order of the caption in the text.

In the revised submission, we have paid attention in ordering the figures in the same way of the caption in the text.

- Page 4: line 104: strange formulation; first it is mentioned that 1 is the spectral radius and then it says: 1 also belongs to the spectrum; this is redundant information

In the revised manuscript we have changed this into “The spectral radius of P is 1; in particular, 1 belongs to the spectrum of P”. However, note that there is no redundancy: the first part does not imply the second one, as it could be that only -1 (and not 1) was in the spectrum of P. Also, the second part does not imply the first one, as if 1 belongs to the spectrum, it does not imply that 1 is also the eigenvalue of largest modulus.

- page 6: line 176: I don't think the general reader knows what "tele-portation" means in the setting of PageRank. Also check spelling: teleportation?

We thank the Reviewer for this comment. In the revised version of the manuscript we have clarified the meaning of teleportation, and we have adopted the recommended spelling without the hyphen symbol.

-page 7: line 268: How is "meeting individuals from the same community" defined? Just related to connectivity or in terms of mobility or in terms of probability?

It is in terms of probability. We now briefly explain how: we first associate each individual with a community. Then, we add edges between nodes (i.e., individuals) with a certain probability: the probability that we add an edge between two individuals of the same community is 19%, while the probability that we add an edge between two individuals belonging to two different communities is 0.1%. Every day (apart from the simulations based on the same fixed scenario), a new (and in principle completely different) graph is generated according to this same procedure.

Obviously, the information used to create the graph (e.g., the community of each individual) is then not exploited by any of the indicators used to decide who should be tested.

- page 8: line 237: "both solutions". Which solutions are you referring to?

We were referring to the possibility of testing individuals either on the basis of the ranking of node degree, or of the proposed Kemeny-based indicator. In the revised version of the manuscript, we have revised this sentence to clarify our meaning.

- page 8: kine 249: "those based on random walks" Do you mean based upon the Kemeny indicator?

We were meaning both the proposed Kemeny-based indicator, and also the “Random Walk Betweenness” indicator proposed by Newman in reference [23]. In the revised version of the manuscript, we have revised this sentence to clarify our meaning.
